# *QuickStats:* Age-Adjusted Suicide Rates,* by Sex and Three Most Common Methods^†^ — United States, 2000–2018

**DOI:** 10.15585/mmwr.mm6909a7

**Published:** 2020-03-06

**Figure:**
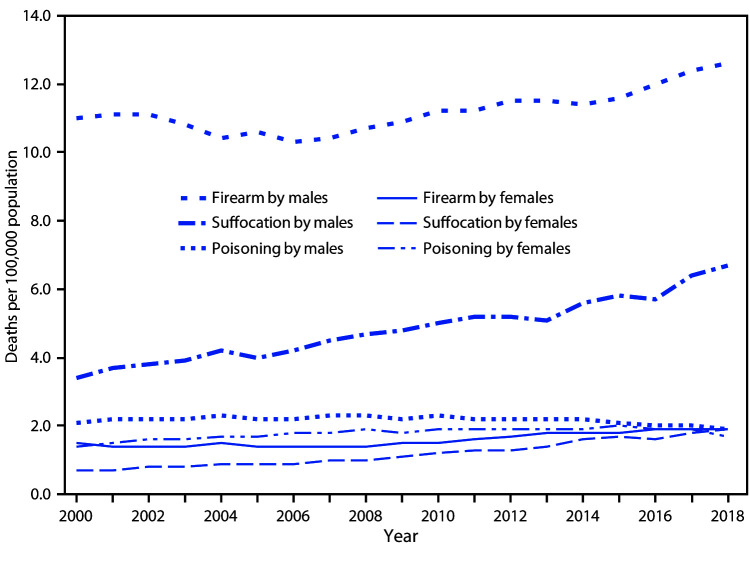
The three most common methods of suicide among males and females during 2000–2018 were by firearm, suffocation, and poisoning. After remaining steady from 2000 to 2006, age-adjusted firearm suicide rates increased during 2006–2018 among males (from 10.3 to 12.6 per 100,000) and females (from 1.4 to 1.9). Suffocation suicide rates among males and females increased steadily during 2000–2018 (from 3.4 to 6.7 for males and from 0.7 to 1.9 for females). In contrast to the other suicide methods, poisoning suicide rates during 2000–2018 initially increased and then declined, from 2.3 in 2010 to 1.9 in 2018 among males and from 2.0 in 2015 to 1.7 in 2018 among females. Throughout the period 2000–2018, suicide rates by all methods were higher among males than among females, with the greatest difference in the rates for suicide by firearm.

**Source:** National Center for Health Statistics, National Vital Statistics System, mortality data. https://www.cdc.gov/nchs/nvss/deaths.htm.

For more information on this topic, CDC recommends the following link: https://www.cdc.gov/violenceprevention/suicide/index.html.

